# Exploring changes in guest preferences for Airbnb accommodation with different levels of sharing and prices: Using structural topic model

**DOI:** 10.3389/fpsyg.2023.1120845

**Published:** 2023-02-17

**Authors:** Kai Ding, Wei Chong Choo, Keng Yap Ng, Qing Zhang

**Affiliations:** ^1^School of Business Administration, Ningbo University of Finance and Economics, Ningbo, China; ^2^School of Business and Economics, Universiti Putra Malaysia, Serdang, Malaysia; ^3^Institute for Mathematical Research, Universiti Putra Malaysia, Serdang, Malaysia; ^4^Faculty of Computer Science and Information Technology, Universiti Putra Malaysia, Serdang, Malaysia; ^5^School of Business, Linyi University, Linyi, China

**Keywords:** online user behavior, text analytics, topic modeling, sharing economy, Airbnb

## Abstract

**Introduction:**

Online reviews have become an important source of information for investigating customers’ consumption experiences in academic studies. In the context of sharing economy-based accommodation, various studies have been conducted to investigate the user experience of Airbnb by analyzing online reviews; however, most previous Airbnb studies had focused on analyzing the user experience of Airbnb at a holistic level without distinguishing the accommodation attributes of Airbnb. Therefore, this article aimed to investigate how the preferences revealed by Airbnb users in online reviews vary across Airbnb listings with different levels of sharing and price ranges.

**Methods:**

This study analyzed 181,190 online reviews under Airbnb listings in Kuala Lumpur, Malaysia, using the structural topic model (STM).

**Results:**

This study identified 21 topics related to Airbnb service and product attributes.

**Discussion:**

The findings show that Airbnb users who stay at *entire property* are more concerned with the hedonic value of their stay, while those who *stay at shared* property are more concerned with the utilitarian value. The purposes of the host–guest interaction were also found to differ between these two types of Airbnb accommodations. Regarding the effect of listing prices on users’ preferences, findings reveal that those staying at lower-priced rooms were more concerned about the convenience of exploring the surrounding area, while those who stayed at higher-priced rooms were more concerned about the surrounding environment and the interior facilities of the property.

## 1. Introduction

With the rapid development and popularity of the Web 2.0 era, an increasing number of people are more inclined to express their thoughts and interact with each other through the Internet on social platforms. Compared with product descriptions published by merchants, online customer reviews are the expressions of consumers’ emotions after their real experiences with the products, so they are more trustworthy and persuasive (Ham et al., 2019). Especially, for experiential products, such as accommodation products, which are one of the most typical experiential products, online customer reviews have been found to have a significant impact on the consumption behavior of potential customers (Chen and Farn, 2020).

The peer-to-peer (P2P) accommodation platforms, driven by technological advances (Gupta et al., 2019), have emerged as new marketplaces to exchange unused accommodation capacity. P2P accommodation, as an important component of the sharing economy, has experienced rapid growth in the past few years. A statistic from the World Economic Forum (2017) shows that the global hospitality industry’s annual revenue from short-term rentals is expected to an increase from 7 to 17% by 2025, resulting in an annual migration of US$8 billion in profits from the traditional hospitality industry to the P2P accommodation market sector. Although COVID-19 had a significant impact on the development of P2P accommodation, the P2P accommodation business will soon obtain new opportunities for growth again in the context of the gradual opening of global travel (Pitrelli, 2022). In particular, for some countries that are more dependent on the tourism economy, the development of P2P accommodation plays an increasingly important role in contributing to the local tourism economy (Lee et al., 2020).

To support the sustainability of P2P accommodation businesses, many studies have been conducted to understand guest preferences, with the most prevalent study on Airbnb. Airbnb is the leading P2P accommodation platform that has operated its business in more than 220 countries and has 6 million listings worldwide (Airbnb Statistics, 2022). Researchers investigated the accommodation experience of Airbnb users from different perspectives, such as service quality perceptions (Ju et al., 2019; Ding et al., 2020; Zuo et al., 2022) and customer satisfaction (Priporas et al., 2017; Xu, 2020). In previous studies, online customer reviews have been widely used as a source of data to understand Airbnb user preferences, which provides a more effective and comprehensive solution for gaining insight into the customer experience (Ibrahim and Wang, 2019).

Although many scholars have conducted research on the user experience of Airbnb, most previous studies have only focused on the Airbnb service as a whole without considering its characteristics, such as room type and listing price. Therefore, the generalized findings of previous studies may not reflect the true perceptions of all Airbnb users because they evaluate Airbnb’s service and product attributes differently (Xu, 2020). By taking advantage of big data techniques, this study intends to extend previous studies by examining Airbnb users’ emphasis on different service attributes in different types of rooms and properties with different price ranges. Considering that sharing is a unique feature of P2P accommodation, this study divides Airbnb’s accommodation into *shared property* and *entire property*. Two research questions are developed as follows:

1. How the importance Airbnb users place on service attributes varies with the degree of room sharing?
2. How the importance Airbnb users place on service attributes varies across properties in different price ranges?

To answer these questions, the authors collected Airbnb reviews under listings from Kuala Lumpur, Malaysia, and structural topic model (STM; Roberts et al., 2014) was used to identify important insights hidden in these unstructured textual data. This study provides valuable comparative insights into Airbnb users’ lodging experience by analyzing data collected in a developing country, as most Airbnb studies were conducted in developed countries and less attention has been paid to developing countries. In addition, this study adds new knowledge about the behavior of Airbnb users. Notably, this is the first study to examine the impact of the listing price on Airbnb users’ preferences, providing a reference for future research. From the perspective of methodology, this study employed a novel method (STM) to analyze online review data. This study shows the viability of including customized metadata in the topic model, which can aid in uncovering hidden information, in contrast to prior studies that simply integrated existing metadata in the STM.

The rest of this study is organized as follows. The “Literature review” section describes related studies on customer reviews, Airbnb, and methods for text data analysis. The “Methodology” section describes the detailed data analysis methods and procedures. The “Results” section presents the results of the textual data analysis. The “Conclusion” section presents the discussions of the main findings, as well as theoretical and managerial implications, and concludes the study.

## 2. Literature review

### 2.1. Customer reviews

Customer reviews have been used as a valuable source of information to determine customer preferences. This is especially prevalent in the age of Internet 4.0. Many customers would like to post their consumption experience online, which contains detailed information about customer preferences (Wang et al., 2020). Online reviews are also a source of information for customers to form their expectations of products and services (Xu and Li, 2016). According to the expectation confirmation theory (Oliver, 1980), consumers make predictions about the timing of purchase transactions, generate expectations, and form opinions about the performance of the service. Perceptions of performance are directly influenced by pre-purchase or pre-acceptance expectations and, conversely, directly influence the negation of beliefs and post-purchase or post-acceptance satisfaction. Examining customer reviews can provide an effective solution to anticipate customer expectations revealed in the reviews, which can support the development of strategies to improve customer satisfaction.

In the context of Airbnb, many studies have been conducted to draw insights from customer experience data. Ding et al. (2020) used the STM to explore the key service quality attributes of Airbnb through the analysis of 242,020 online customer reviews. The findings revealed 22 service attributes that Airbnb users frequently wrote about in their reviews. This study also differentiates the preferred accommodation experiences of Airbnb users by nationality and examines temporal changes in attention to extracted service quality attributes, with results showing the unique preferences of Malaysian and international Airbnb users, as well as patterns of change in selected service attributes over a 5-year period. Ju et al. (2019) investigated the key service quality attributes of Airbnb and examined the asymmetric effects of these Airbnb service quality attributes on user satisfaction. By conducting a content analysis of collected Airbnb reviews from four major cities in the United States, this study determined four key topics, namely, “host,” “room/house,” “location,” and “neighborhood.” From the perspective of customer satisfaction, Ding et al. (2021) explored the sources of satisfaction and dissatisfaction with Airbnb services using Airbnb reviews as a data source. This study analyzed Airbnb reviews generated in 12 different cities, and the results show the different components of satisfiers and dissatisfiers in Airbnb services. Tangible attributes are the major sources of dissatisfaction, while interaction experience with Airbnb hosts is the major source of satisfaction. Serrano et al. (2021) investigated Airbnb green users’ preferences and sustainable attitudes by analyzing online Airbnb reviews. This study employed the latent Dirichlet allocation (LDA) topic model to discover hidden service attributes from selected reviews. The following six latent aspects that are associated with Airbnb green users were identified: amenities, sustainability, experience, accommodation, host, and location.

### 2.2. Airbnb accommodation

Airbnb has four different accommodation options, namely, the entire room, private property, shared room, and hotel room, which can cater to the needs of different customers. Various types of rooms can have different performance results with different product and service attributes, which can lead to those consumers staying in these different types of rooms and may evaluate these attributes differently (Xu, 2020). Regarding the research on different types of accommodation on Airbnb, Abdar and Yen (2017) found that the perceived importance of different types of Airbnb properties differs in different countries, which indicates the different preferences of Airbnb users. Lutz and Newlands (2018) found that customers who travel alone or with family or friends prefer to share a room, while those who travel with a partner prefer an entire home. Those traveling with friends and family, in contrast, opt for budget options that allow them to share rooms on their own. In addition, environmental factors also affect customers’ choices of property types. More specifically, guests who are uncomfortable with environmental issues such as dust and hair tend to avoid sharing rooms. In contrast, guests who are uncomfortable with human interaction tend to prefer to stay in a full home where they are not in contact with the host or other guests. More recently, Bresciani et al. (2021) investigated the role of Airbnb property types when choosing Airbnb services during the pandemic. Due to the concern of health risks, Airbnb users were found to be more in favor of types of rooms that can guarantee physical distance. There is no doubt that previous studies have found that Airbnb users who stayed at different types of accommodations have varying practical needs. However, there are few studies that differentiate accommodation types to study users’ needs, and this study intends to fill this gap.

### 2.3. Accommodation price

In the accommodation industry, price is one of the most important factors influencing customers’ expectations of service (Knutson et al., 1993; Ye et al., 2014). However, in the context of P2P accommodation, less research has focused on how customers’ emphasis on the attributes of Airbnb accommodations varies across the price range. In the traditional hospitality industry, Knutson et al. (1993) investigated customers’ expectations for service quality in hotels with different price ranges, namely, economy, mid-price, and luxury hotels. This study found that travelers’ expectations increased when they moved up the hotel price scale. For instance, travelers have higher expectations of the service quality dimensions of responsiveness and tangibles in luxury hotels. Ye et al. (2014) examined the impact of hotel prices on customers’ perceptions of service quality and value through the analysis of online traveler reviews. The findings of this study show that price has a positive impact on the perceived quality of hotel guests but has a negative impact on the perceived value, which signifies the varying expectations of customers living in hotels with different price ranges. Chiu and Chen (2014) examined the relationship between price and hotel service quality based on signaling theory. The findings of this study suggest that higher prices may signal higher service quality. Rhee and Yang (2015) examined the differences in the importance of hotel attributes across hotels using hotel star ratings as a segmentation criterion. This study focused on six hotel attributes, namely, value, location, sleep quality, room, cleanliness, and service, and the results showed that customers valued different hotel attributes differently in hotels with different star ratings. Kim et al., 2019 compared tourists’ perceptions of hotel attributes in luxurious and economical hotels. The results indicate that tourists staying at luxury and budget hotels do not place the same level of importance on hotel attributes. For instance, only luxury hotel users were found to be concerned with room comfort and decoration. It can be clearly seen that customers’ expectations of hotels in different price ranges are different, and as prices increase, so do customers’ service requirements. In the traditional hotel industry, the grading of the hotel star rating generally reflects the price range of the hotel. More specifically, the higher the “star rating,” the higher the price, and the lower the “star rating,” the lower the price (Henley et al., 2004). However, for P2P accommodations, there is no unified index for classifying price levels, so it is relatively difficult to discover the preferences of P2P accommodation users at different prices. To fill a gap in the current research on the impact of Airbnb listing prices on customer service perceptions, this study used a big data approach to understand the preferences of Airbnb users at different price levels.

### 2.4. Textual data analysis

Due to the fact that processing a large quantity of online customer reviews is far beyond the capability of human coding, hence, many researchers applied text mining techniques to extract valuable information from those unstructured textual data. To identify the subjective information in text, sentiment analysis is a common method. In consumer research, sentiment analysis refers to the process of identifying various emotions about a product or service in a text, such as positive, negative, or neutral impressions. Sentiment analysis is often used to measure customers’ subjective feelings, such as customer satisfaction (Ding et al., 2021) and perceptions of service quality (Ju et al., 2019). Since the focus of this study is to discover specific categories of customer needs without considering emotional responses, sentiment analysis is not applied in this study.

For lexical-level analysis, term frequency analysis is one of the most popular methods to analyze customer reviews; this is particularly prevalent in the tourism and hospitality industries. Researchers use term frequency analysis to study the importance of words in a text or group of texts by measuring how often certain words occur (Song and Myaeng, 2012). Even though term frequency-based approaches can provide researchers with a fast solution to draw insights from customer reviews, using this method has many limitations. One of the major limitations is that term frequency analysis only provides a summary of the number of terms without considering the context of the words, which may lead to ambiguity and confusion in the conclusions (Büschken and Allenby, 2016). In addition, the term frequency method can only be applied at the lexical level, as this method cannot capture the semantic relationships between different terms, which leads to an under-exploitation of the value of the customer review data. As for document-level analysis, text clustering is often applied for the purpose of document classification. In the clustering process, a pre-designed algorithm clusters the documents into different groups based on similarity measures. Despite the effectiveness of using text clustering to group textual documents, this method may not be suitable for analyzing customer reviews. This is because each customer review contains multiple aspects or topics about the consumer experience, and it is not enough to assign a document to a specific group, which can lead to the loss of important insights. To address this limitation, topic models are often used in mining customer reviews.

### 2.5. Topic modeling

Topic modeling is a machine learning-based approach that automatically detects topics from textual documents. Although the topic-generation process is automated, human judgment also plays an important role. To illustrate, manual evaluation is usually required to determine the quality of topic models, as relying on quantitative metrics alone can result in several issues, such as the appearance of overlapping topics and poor interpretability. In addition, it is challenging to interpret the meaning represented by a topic based solely on a list of topic words, and manual analysis of representative texts on the topic is required to produce more accurate results. As for the specific topic model, LDA is a topic modeling method commonly used to identify topics in various domains (Blei et al., 2003). LDA is developed from the Probabilistic Latent Semantic Indexing (PLSI) model (Deerwester et al., 1990), which is a three-level Bayesian mixture model based on the bag-of-words assumption over the document-topic-word. LDA also serves as the basis for many extensions. After the LDA model was proposed, many extensions were developed to suit research with different purposes. For example, several extended models were developed to reflect the parameters of document-topic probability distributions and topic-word probability distributions, such as the correlated topic model (Blei and Lafferty, 2007) and the dynamic topic model (Blei and Lafferty, 2006).

**Figure 1:**
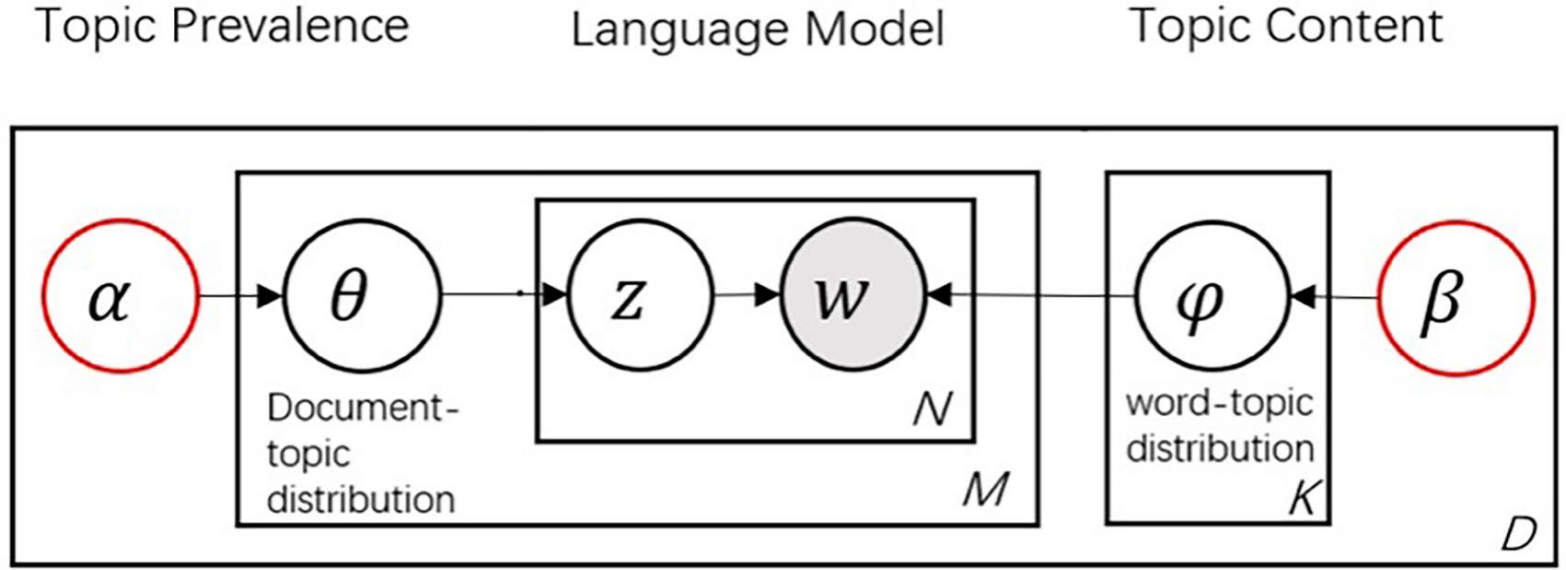
Plate diagram of latent Dirichlet allocation.

Despite the development of various LDA-based models, LDA is still one of the most widely applied probabilistic topic modeling techniques in machine learning. LDA has been applied in consumer research for different purposes, such as customer satisfaction (Ding et al., 2021; Wang et al., 2021), consumption experience (Sutherland and Kiatkawsin, 2020; Zhuo and Wang, 2022), and purchase intention (Sim et al., 2021; Zhang et al., 2021). The underlining assumption of LDA is that a document is composed of words that help determine the topic, and LDA maps the document to a list of topics by assigning each word in the document to a different topic (Hagen, 2018). As for the generative process of LDA, a corpus *D* and pre-defined topic number *K* serve as the main input. The LDA process for review texts is graphically depicted in Figure 1 using plate notation. The steps are described as follows.

The first step is to sample the document-topic probability distribution *θ_i_,* which is generated from the Dirichlet distribution of hyperparameters *α*. The second step is to sample the topic-term probability distribution *φ_k_*, which is generated from the Dirichlet distribution of hyperparameter *β*. As for the third step, topic *z_i,n_* is generated from the topic distribution *θ_i_ of* document *D_i_*, and topic term *w_i_* is generated from the probability distribution of *φ_k_* of topic *z_i,n_*. The output of the LDA model includes document-topic distribution matrix *θ_i_* and topic-term distribution matrix *φ_k_*.

Based on the Dirichlet-multinomial regression topic model of Mimno and McCallum (2012) and the sparse additive generative model of Eisenstein et al. (2011), Roberts et al. (2014) proposed the STM, which is a flexible multi-covariate topic model. Similar to LDA, STM is also a generative model, i.e., the topics (*T*_1_,*T*_2_,…,*T*_k_) are defined as document-level lexical items (*w*_1_,*w*_2_,…, *w*_n_), and each document (*D*_1_, *D*_2_, …, *D*_d_) can be composed of multiple topics (Blei, 2012).

**Figure 2:**
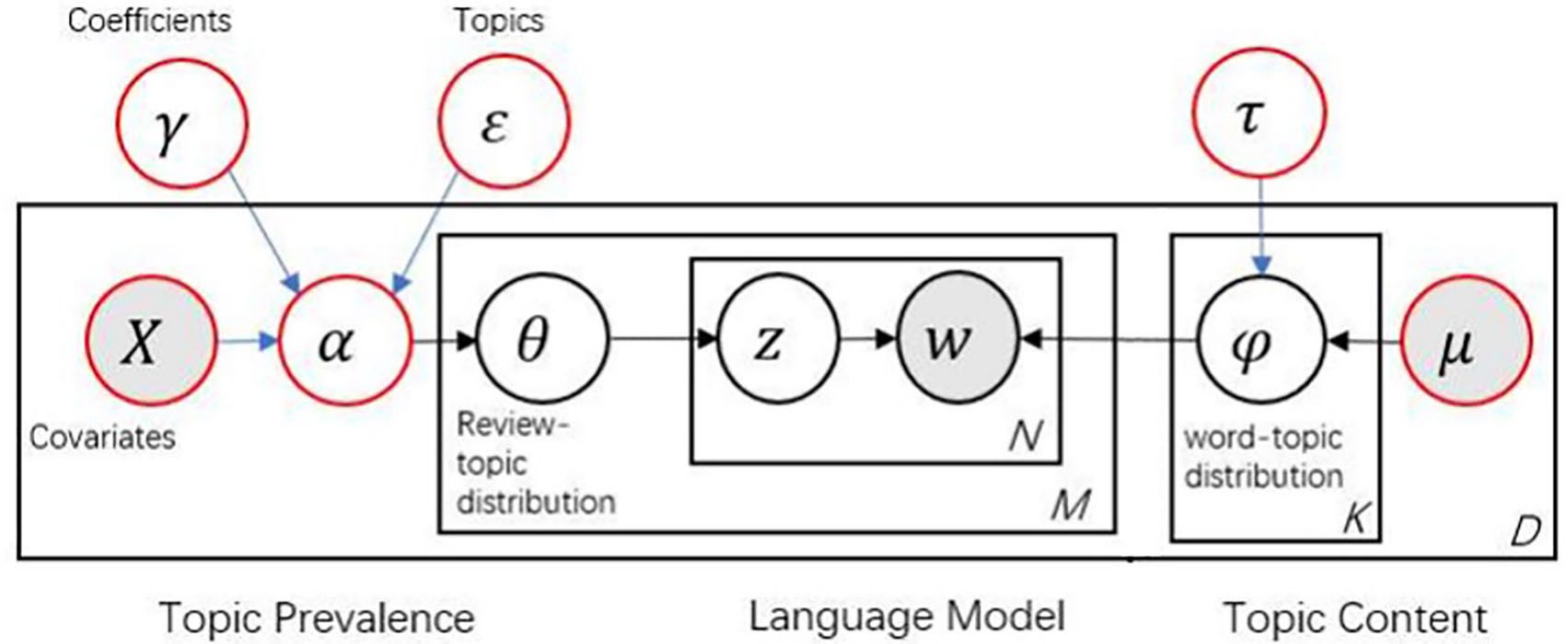
Plate diagram of structural topic model.

Structural topic model differs from LDA in that the STM allows the inclusion of several document-level covariates, such as *X_d_*, in the document generation process, and the incorporated covariates can be either continuous or discrete. Figure 2 shows the document generation process. The model of STM assumes the following generative process for each document *D* in the corpus:

- Generating document topics in a general linear model based on document-level covariates *X_d_*,

(1) $${\vec{\theta }}_{d}|X_{d}\gamma ,\sum ∼\mathrm{Logistic}\left(\mu =X_{d}\gamma ,\sum \right)$$

- Using the log frequency distribution (*m*), topic-specific deviation (*K_k_*), covariate-specific deviation (*K_c_*), and its interaction deviation *K*_*i* = (*kgd*)_, a word distribution that represents each topic (*k*), which is modeled as follows:

(2) $$\beta_{d,k}\propto \exp\left(m+K_{k}+K_{gd}+K_{i=\left(kgd\right)}\right)$$

- Terms in each document *n* are assigned to topics based on a document-specific topic distribution as follows:

(3) $$z_{d,n}|{\vec{\theta }}_{d}∼\mathrm{Multinomial}\left(\vec{\theta }\right)$$

- An observed term generated from the selected topic is given as follows:

(4) $$w_{d,n}|z_{d,n},\beta_{d,k=z}∼\mathrm{Multinomial}\left(\beta_{d,k=z}\right)$$

The most significant improvement of STM over LDA is the introduction of document-level structural information to influence the topic prevalence and topic content, thus highlighting the applicability of examining how covariates affect the text content. Topic prevalence and topic content can be represented as functions of document metadata. Topic prevalence indicates how much of a review is related to a topic, and topic content is represented by a list of words in the topic. Taking the objectives of this study into consideration, STM is more appropriate for this study because it has the advantage of incorporating covariates into the thematic model, which allows the researchers to examine how Airbnb users’ preferences change across different types of rooms and at different price levels.

## 3. Methodology

### 3.1. Dataset description

**Figure 3:**
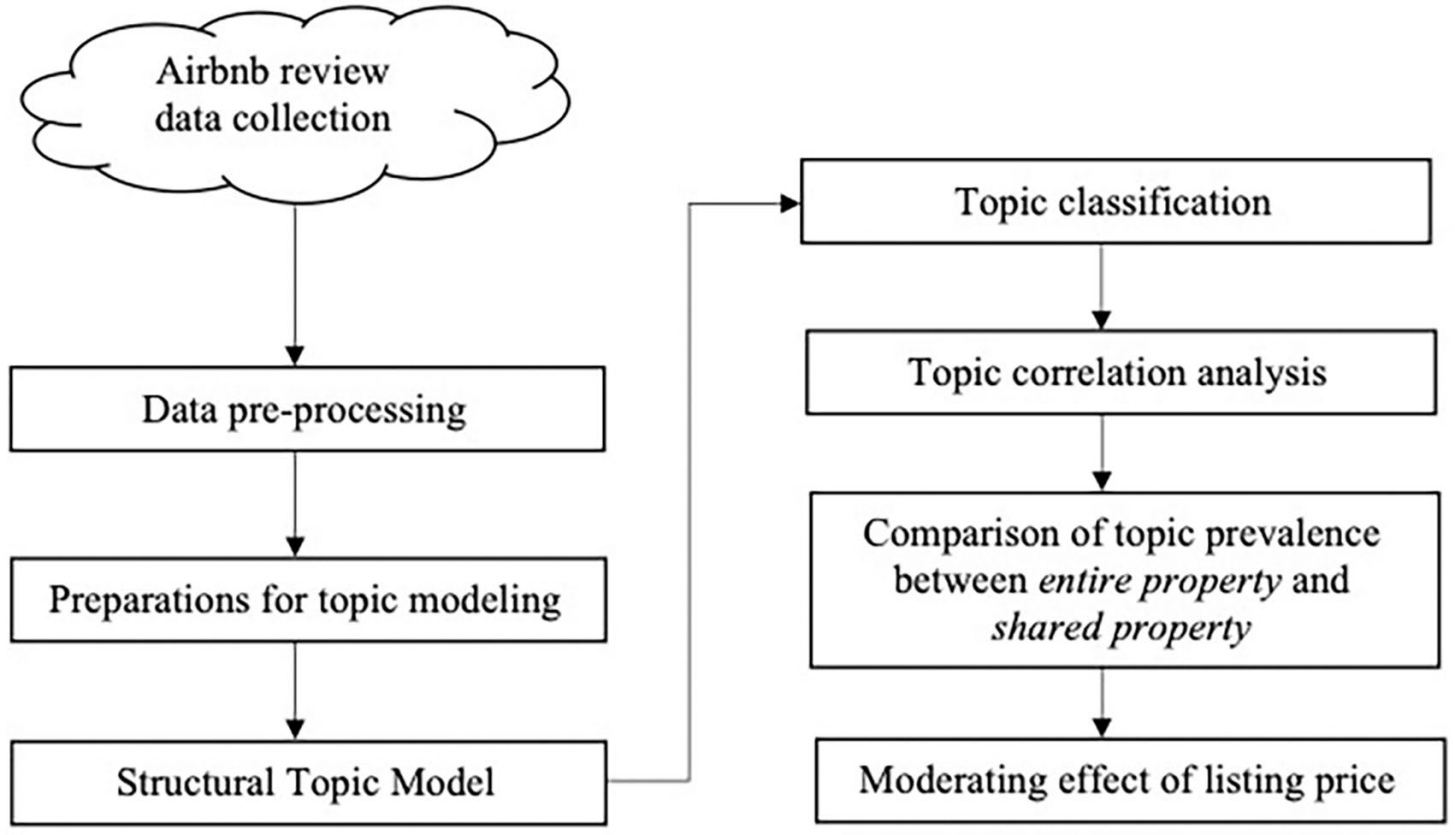
The research procedures.

The Airbnb review dataset was acquired from AirDNA, a data analytics company for vacation rental entrepreneurs and investors. AirDNA has served as a data provider for several Airbnb studies (e.g., Gibbs et al., 2018). Based on the objectives of this study, five variables are kept for the following analysis, including property ID, review date, listing type, average daily price (unit: Malaysian ringgit), and reviews. After filtering out non-English reviews, a total of 181,190 reviews were generated in Kuala Lumpur during the period of January 2015 to January 2020. Due to the domestic movement control and the policy that foreign tourists cannot enter the country in Malaysia, the rent for Airbnb in Malaysia has experienced an abnormal decline during this period, which could affect the accuracy of the analysis of the impact of price on user preference. Therefore, data generated during the pandemic were excluded. Figure 3 shows the detailed research procedures.

### 3.2. Text pre-processing

Text pre-processing is performed using R programming. First, comments with less than 50 words are removed; numbers and punctuation are eliminated using the “tm” package; words are converted into lowercase letters, stop words are removed by using the SMART dictionary (e.g., “the,” “and”), and then custom stop words (e.g., system-generated words) are removed. To reduce the dimensionality of the corpus, all words were converted into their stems. For example, both “work” and “working” were reduced to “work.” To filter out noise words, words with fewer than two letters that appear in less than 1% of the total corpus are removed. Common bigrams are converted into a single word (e.g., “air conditioner,” to “aircond,” “wi fi,” to “wifi”). After pre-processing, there are 133,166 reviews for the topic modeling analysis.

### 3.3. Covariates setup

Two covariates are included in the topic model, namely, listing type and listing average price. The listing type is divided into two groups based on the level of sharing, namely, *entire property* (i.e., entire room) and *shared property* (i.e., private property, shared room, and hotel room). The price range is based on the average daily rate of the listing, represented by numbers 1, 2, 3, and 4. For *entire property*, the four price ranges are less than RM164, RM164 to RM228, RM228 to RM324, and more than RM324. For *shared property*, the four price ranges are less than RM65, RM65 to RM100, RM100 to RM174, and more than RM174.

### 3.4. Topic number estimation

Before fitting the STM, the optimal number of topics needs to be determined. Semantic coherence is an important metric for evaluating topic models. Semantic coherence is a metric related to pointwise mutual information, where the core idea is that, in a semantically coherent model, the words most likely to occur in a topic should co-occur in the same document (Belwal et al., 2021). The topic models that achieved better clustering results were found to have more consistent semantics within the same topic and distinct semantic segmentation features between topics (Airoldi and Bischof, 2016). Mimno et al. (2011) proposed a semantic coherence index to measure the quality of each topic model as follows:


(5) $$C_{k}=\overset{M}{\underset{i=2}{\sum }}\overset{i-1}{\underset{j=2}{\sum }}\ln\left(\frac{D\left(v_{i},v_{j}\right)+1}{D\left(v_{j}\right)}\right),$$

where *D*(*v_i_*) denotes the number of occurrences of the term *v_i_* in a document, and *D*(*v_i_*,*v_j_*) denotes the number of occurrences of both words *v_i_* and *v_j_* in a document. Using the semantic coherence metric can achieve better performance in determining topics with higher quality, and at the same time, evaluating topic quality based on word co-occurrence is also consistent with the logic of subjective evaluation of topic quality by humans. However, using the semantic coherence metric alone to determine the optimal number of topics may result in some topics being dominated by a number of common words, making it difficult to distinguish each topic (Ling et al., 2021). To avoid such bias, the FREX value proposed by Airoldi and Bischof (2016) is used to measure the exclusivity of terms in different topics. The exclusivity measure is calculated by summing the frequency indicator scores of the most frequently occurring words in a model, and it can be used to distinguish this topic from other topics. The FREX value is modeled as follows:

(6) $${\mathrm{FREX}}_{k,v}={\left(\frac{\omega }{F\left(\beta_{k,v}/\left(\sum_{j=1}^{K}\beta_{j,v}\right)\right)}+\frac{1-\omega }{F\left(\beta_{k,v}\right)}\right)}^{-1},$$

where *k* is the *k*-th topic, *v* is the term under consideration, *β* is the word distribution of the *k*-th topic, *ω* is the prior used to impose exclusivity, and the weight ω is set to be 0.7 by default. In line with Hu et al. (2019) and Korfiatis et al. (2019), two statistical criteria were used to determine the optimal number of topics in this study, namely, semantic coherence and exclusivity.

**Figure 4:**
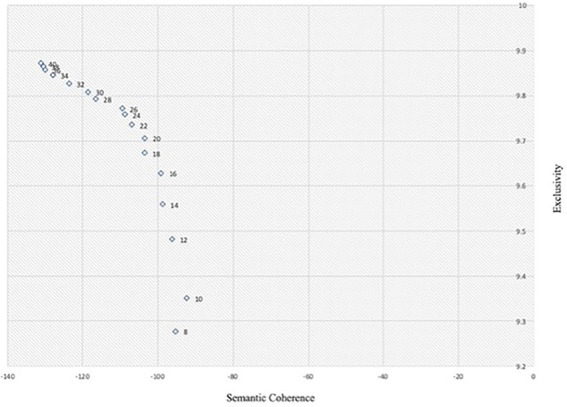
Semantic coherence and exclusivity of different topic model solutions. For the semantic consistency metric, the closer the value is to 0, the better. For the exclusivity metric, the closer the score is to 10, the better.

Figure 4 demonstrates the performance of different topic solutions, which reveals that topic models achieve comparatively better scores in both coherence and exclusivity in the range of 18–22 topics. To identify a suitable number of topics in this range, a manual evaluation is conducted to assess the interpretability of topics generated by topic models with different topic numbers. After evaluation, the authors selected the 21-topic model that generates topics with good interpretability and fewer overlapping topics in this topic solution.

## 4. Results

### 4.1. Topic summary and validation

**Figure 5:**
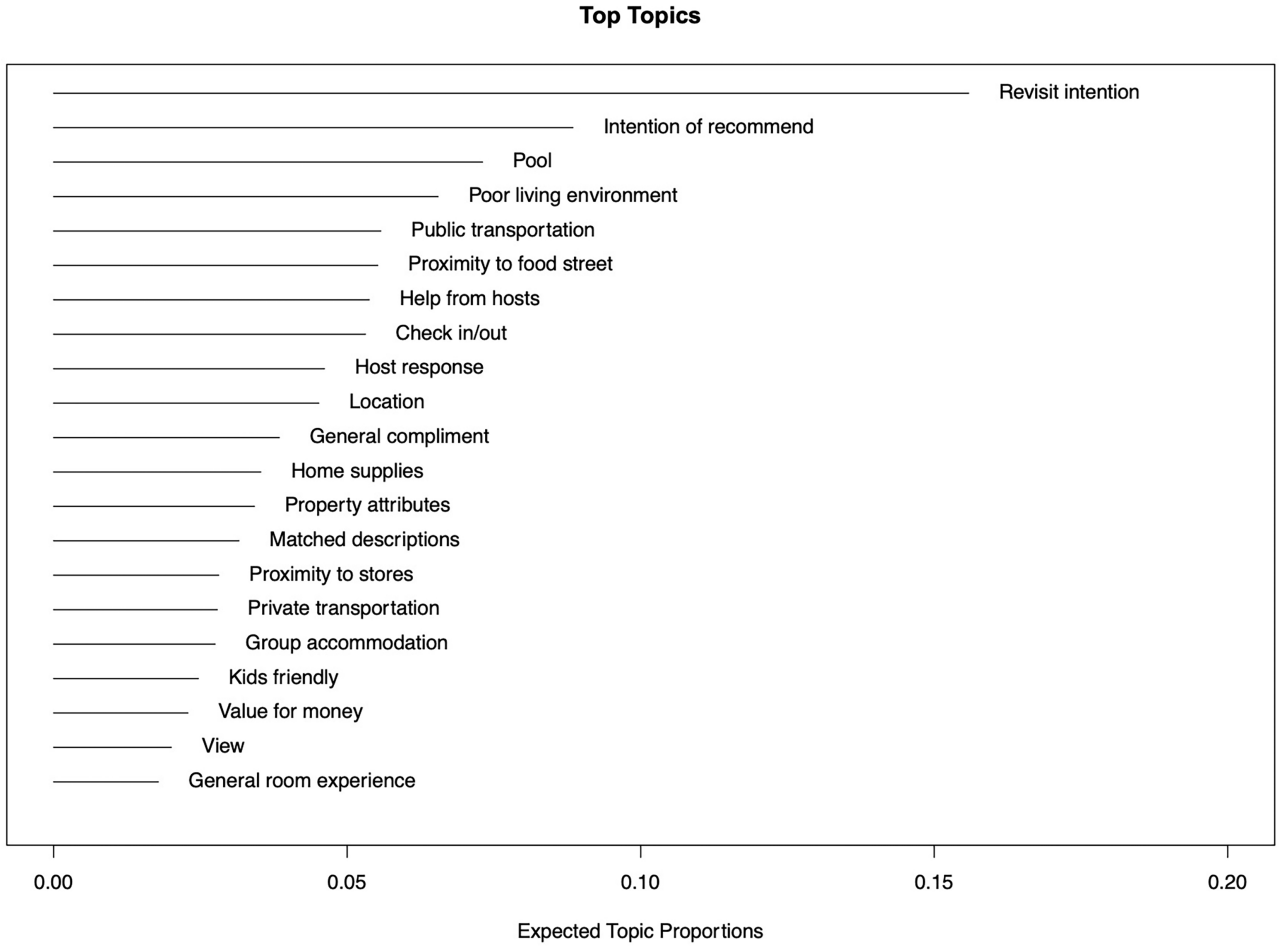
Ranking of topics by proportions.

Table 1 shows the result of the STM, a topic model with 21 topics, 133,166 documents, and a 25,036-word dictionary. The topic labeling process is based on the evaluation of top FREX words that are able to differentiate each topic. The name of each topic was chosen with reference to previous Airbnb studies, all researchers were involved in choosing the appropriate name for each topic, and the name was confirmed when all researchers agreed on it. After confirming the topic names, the authors examine the top 20 representative reviews of each topic to verify the appropriateness of the selected names. Figure 5 shows the ranking of topics in terms of their proportions in the corpus, which reveals the relative importance of each topic. Table 1 presents the classification of extracted topics from the corpus, and Table 2 shows the most representative text of each topic. Consistent with Guo et al. (2017), the extracted topics from the STM were classified into five dimensions, namely, facilities, service, location, value, and general experience.

**Table 1:** Topic classification.

Dimension	Topic no.	Topic label	Top 7 FREX words
Facilities	10	Property attributes	complex, fulli, equip, furnish, appoint, brand, secure
	12	Pool	infin, rooftop, roof, armin, infinit, skylin, appart
	15	Poor living environment	smell, loud, cockroach, bug, broken, smoke, drain
	19	Group accommodation	hous, din, landlord, ezer, suitabl, gather, onn
Service	1	Home supplies	shampoo, iron, deterg, dryer, soap, park, utensil
	2	Help from hosts	tip, gave, care, tour, effort, smile, warm
	6	Check in/out	process, self, instruct, check, earlier, procedur, checkout
	16	Host response	respond, queri, quick, prompt, question, respon, enquiri
	18	Kids friendly	kid, dash, play, cat, fun, game, toy
Location	3	Private transportation	driver, app, taxi, cab, klia, traffic, grab
	4	View	menara, bukit_nana, twin_tow, see, face, overview, overlook
	7	Public transportation	public, sunway, mid_valley, transport, lrt, gateway, ktm
	11	Location	explor, thorough, ideal, short, heart, attract, centr
	13	Proximity to stores	everyth, kelli, need, daryl, downstair, anyth, store
	14	Proximity to food street	alor, jalan, berjaya, time_squar, changkat, pavilion, distanc
Value	9	Intention to recommend	valu, high, money, excel, communic, recommend, great
	20	Value for money	star, penni, five, rate, exceed, hotel, deserv
General Experience	5	Matched descriptions	exact, pictur, forward, shown, photo, look, home
	8	General compliment	ever, best, regret, airbnb, second, john, nikola
	17	Revisit intention	come, back, definit, cozi, comeback, recomend, futur
	21	General room experience	good, over, room, also, enough, clean, night

**Table 2:** Representative text of the topic.

Topic name	Representative text of the topic
Home supplies	The location is good, easy to access, quite at night. Hot water is available. Wifi is fast. No tv cable but they provide dvd player. Kitchen amenities are complete enough (induction stove, microwave, electric kettle, refrigerator) but no rice cooker. Kitchen utensils are available, except cutting knife (only steak and bread knife). There is no kitchen ingredients at all. 7–11 just in front of the building.
Help from hosts	Praveena was very friendly and helpful. She offered to pick us up from the airport which made the journey a lot easier.
Private transportation	Great location, just few minutes taking grab car to kl central, but grab system map cannot find this place, so please get your grab driver search the location at Google map instead of using the existing grab map.
View	The view from the living room, balcony and bedroom is wonderful. You can see two towers of Petronas and KL. There is a dedicated elevator for this room. There is a bus stop in front of you and it is convenient to go to KLCC in a short time with bus No. 302.
Matched descriptions	Clean and comfortable environment. Interiors and furniture fittings are exact match with the description and pictures provided. Nice host, provide clear guidance to entrance and car park.
Check in/out	The unit was not ready when I arrived at 3 pm plus, called up host and said will send cleaner immediately but cleaner arrived by 7 pm. After long hours waiting host said will refund for half a day fee and changed to the other unit. We left the unit to attend dinner without proper resting, it spoiled the whole mood. And I still have not received my refund at the point of time of writing this review. Please be advise to call the host before go in.
Public transportation	Nice and clean, the place is near to a mall, near KTM train station if you are going to Batu caves and also near in LRT station060.
General compliment	Our 5th stay with Edwin and Nani. Great experience as always. Being booked airbnb for more than 100 times, I can say that I’m a very experienced airbnb guest-:). I had great time here that’s why I’m keeping come back. Not to mention the condo located in one of the best neighborhood in KL.
Intention to recommend	Highly recommended. A great host and a great apartment, sparkling clean, modern and in an excellent location.
Property attributes	The apartment is very lovely and comfortable. Everything is provided and kitchen is very well equipped. The building security is excellent and there is a gym, pool and spa in the building which was lovely. We were extremely comfortable.
Location	Great location, quiet yet accessible to many places. Comfy and lovely stay for small group. Thanks!
Pool	Room is very nice with amazing view. The swimming pool on roof is incredible with beautiful views over the city. Thanks!
Proximity to stores	Love the environment! Cafe, groceries store and convenience store are just right inside the building. Will definitely stay there again.
Proximity to food street	Place was clean. About 10 min walk to Bukit Bintang street. Nearby has lots of bars and cafe. There is a stretch of local food outdoor eatery just 5 min walk away.
Poor living environment	Did not sleep for whole night, mattress for master bedroom is too lumpy, not really suitable for sleeping. Blankets for double decker bed are not warm enough due to aircond remote faulty. One of the remote is faulty, where the temperature is unreadable, and another remote has dead batteries. Very disappointed.
Host response	Olivia is very quick to reply to your contact. Quick to help answer any questions. And her place is well kept!
Revisit intention	The place was good! Cozy, comfort and good for family stay. Host very helpful and friendly. Definitely will come back someday
Kids friendly	Chris was accommodating to our early check in request. The apt was clean with comfortable beds, good facilities—pool and child friendly play room. Strategically located within walking distance to shopping malls and eating places—Pavilion and Bkt Bintang area. Chris provided helpful tips to explore round KL.
Group accommodation	Good for a group of people to stay and have bonding time. A big living room that can fit more than ten people.
Value for money	For the price we paid, I think we were better off staying at a five star hotel instead.
General room experience	The room is pretty decent and it has a stunning view of KL TOWER at night. The bed and addition mattress were comfortable and the whole place was well kept and it is in good condition. The host is also considerate and allowed us to check in late where we had the chance to meet Helmi. Overall, I would highly recommend this place to those who wants to stay in Kuala Lumpur.

The comments in the table are taken directly from the Airbnb dataset, and grammatical and/or typographical errors have not been amended.

### 4.2. Topic correlation analysis

**Figure 6:**
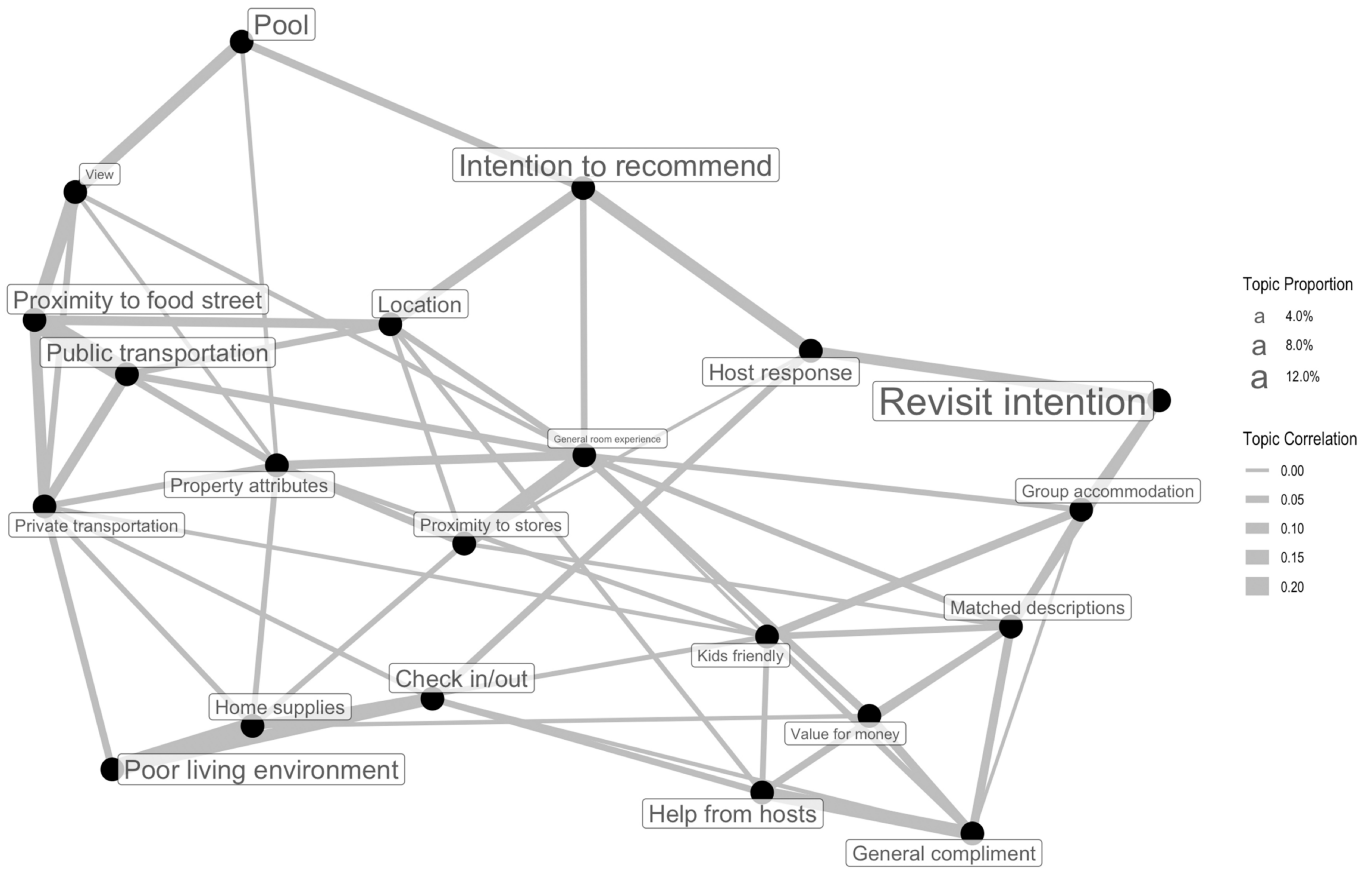
Topic correlation map.

Figure 6 shows the estimated correlations among topics. In Figure 6, topics that often co-occur with high probability are connected, and the thickness of the connecting lines reflects the strength of the correlation. The size of the topic label indicates the relative topic proportions; the larger the topic label, the larger the proportion of this topic. Topic correlations shown in Figure 6 reveal Airbnb users’ preferences and valuable insights into generating positive word-of-mouth for Airbnb hosts. For instance, Airbnb users who mentioned the topic *location* also highlighted the short distance to their desired places, such as *proximity to shops* and *proximity to food street.* When Airbnb users mention transportation-related topics, such as *private transportation,* they tend to care more about the convenience of reaching some well-known shopping malls (e.g., Sunway mall, Mid Valley) in Kuala Lumpur. As for generating positive word-of-mouth, Airbnb users are more likely to express their intentions to revisit the comments when Airbnb hosts provide prompt responses, and the property can accommodate their needs for multiple occupancies. In addition, *host response* is also connected with Airbnb users’ intention to recommend, which signifies the importance of providing a timely reply.

### 4.3. Comparison analysis

**Figure 7:**
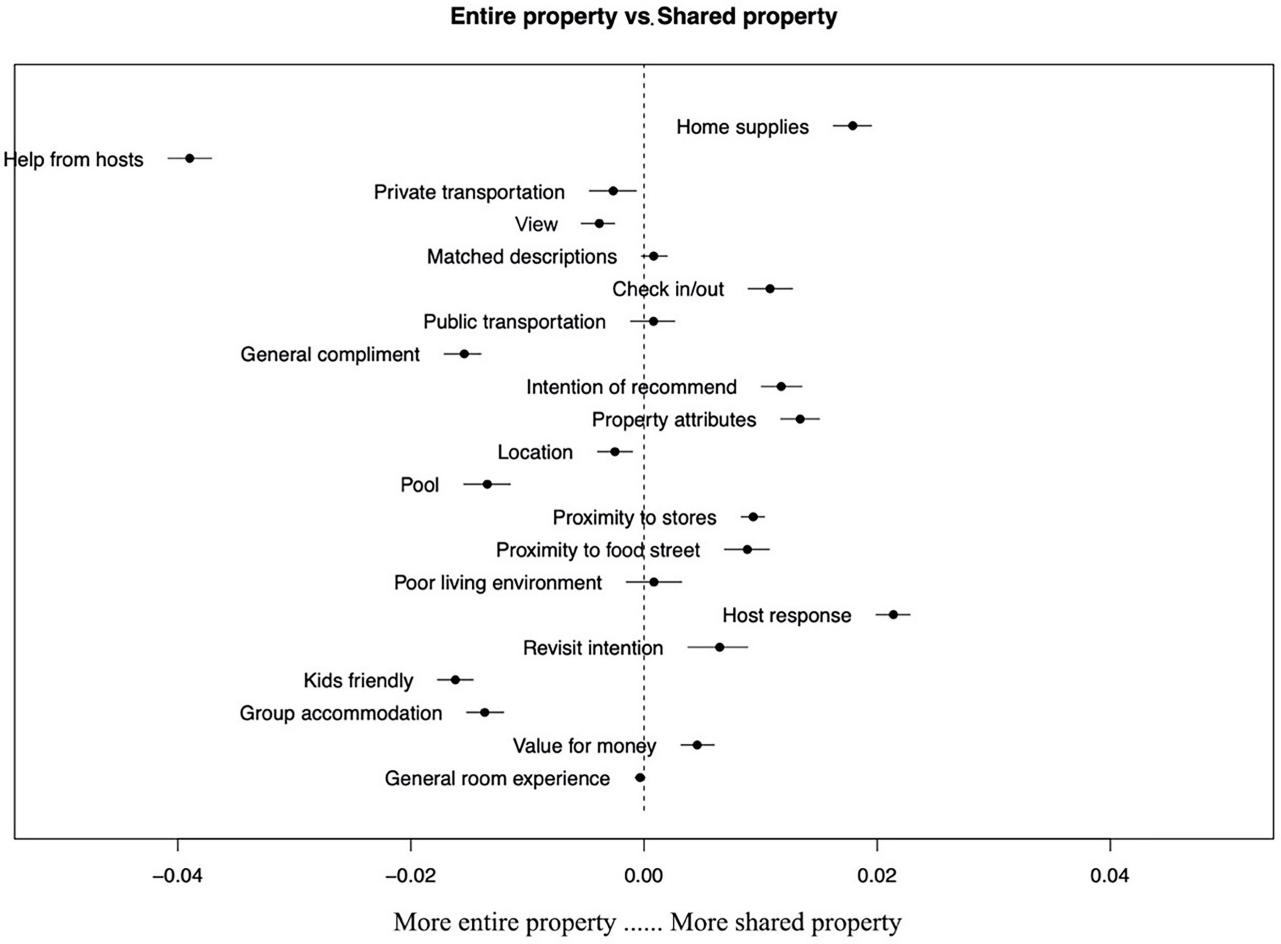
Changes in topic prevalence based on the accommodation type (*entire property* vs. *shared property*).

Figure 7 presents the topics that occur in higher proportions in reviews written by Airbnb users staying at *entire property* and *shared property*. Topics that appeared significantly more in reviews of *entire property* and *shared property* are related to Airbnb hosts. Airbnb users staying at *entire property* often mentioned the help provided by Airbnb hosts, while those staying at *shared property* were more concerned about the Airbnb hosts’ ability to provide timely responses. By comparing the remaining topics, it was found that Airbnb users who stay in *entire property* are more concerned about hedonic value, they usually emphasize properties with pools and properties with views, and they are also more concerned about the experience of their children. As for Airbnb users who stay in *shared property*, they emphasize more utilitarian values such as household items and amenities in the property, and they are also more price sensitive.

### 4.4. Moderating effect of The listing price

**Figure 8:**
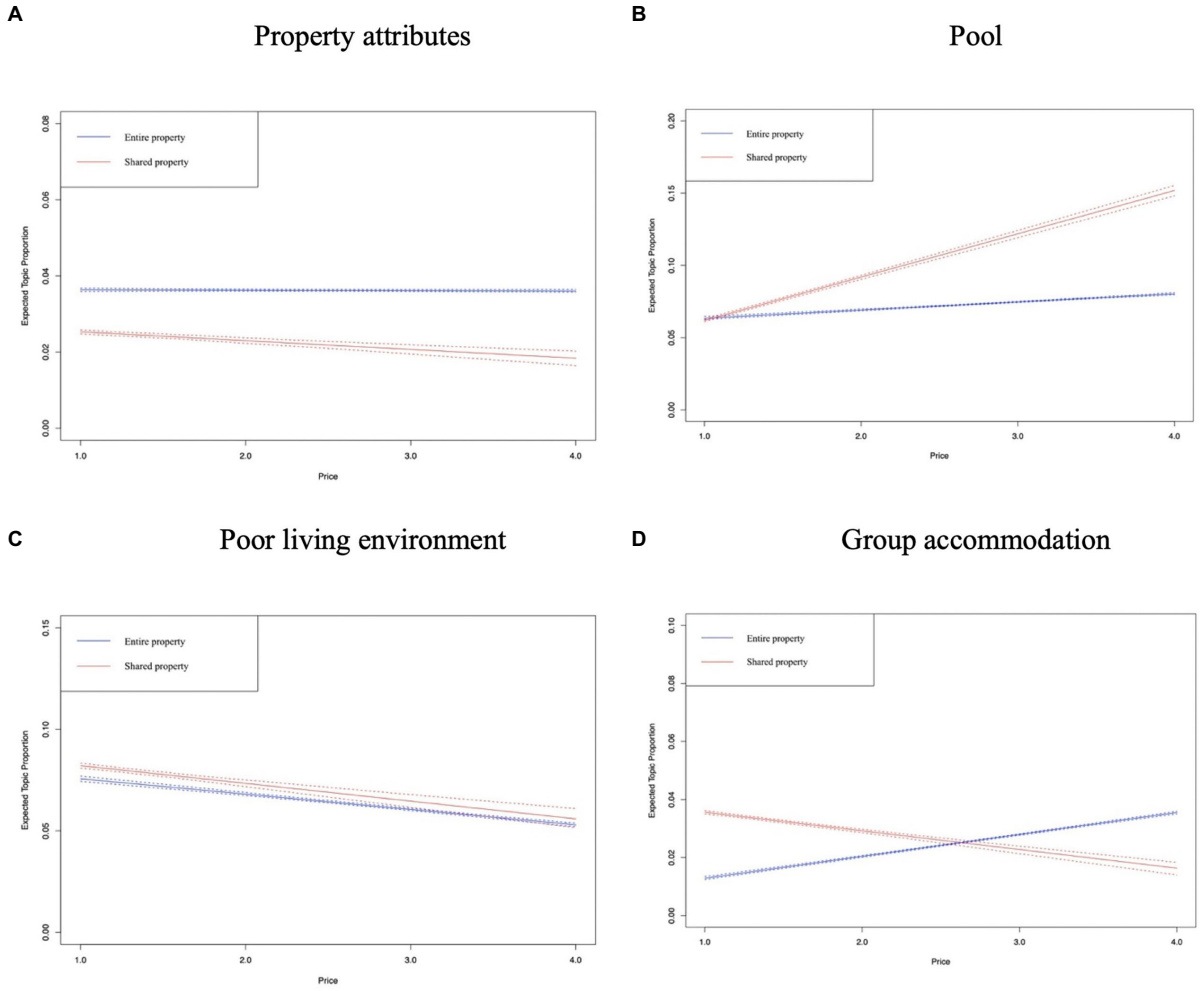
Topics in the dimension of facility. **(A)** Property attributes. **(B)** Pool. **(C)** Poor living environment. **(D)** Group accommodation.

**Figure 9:**
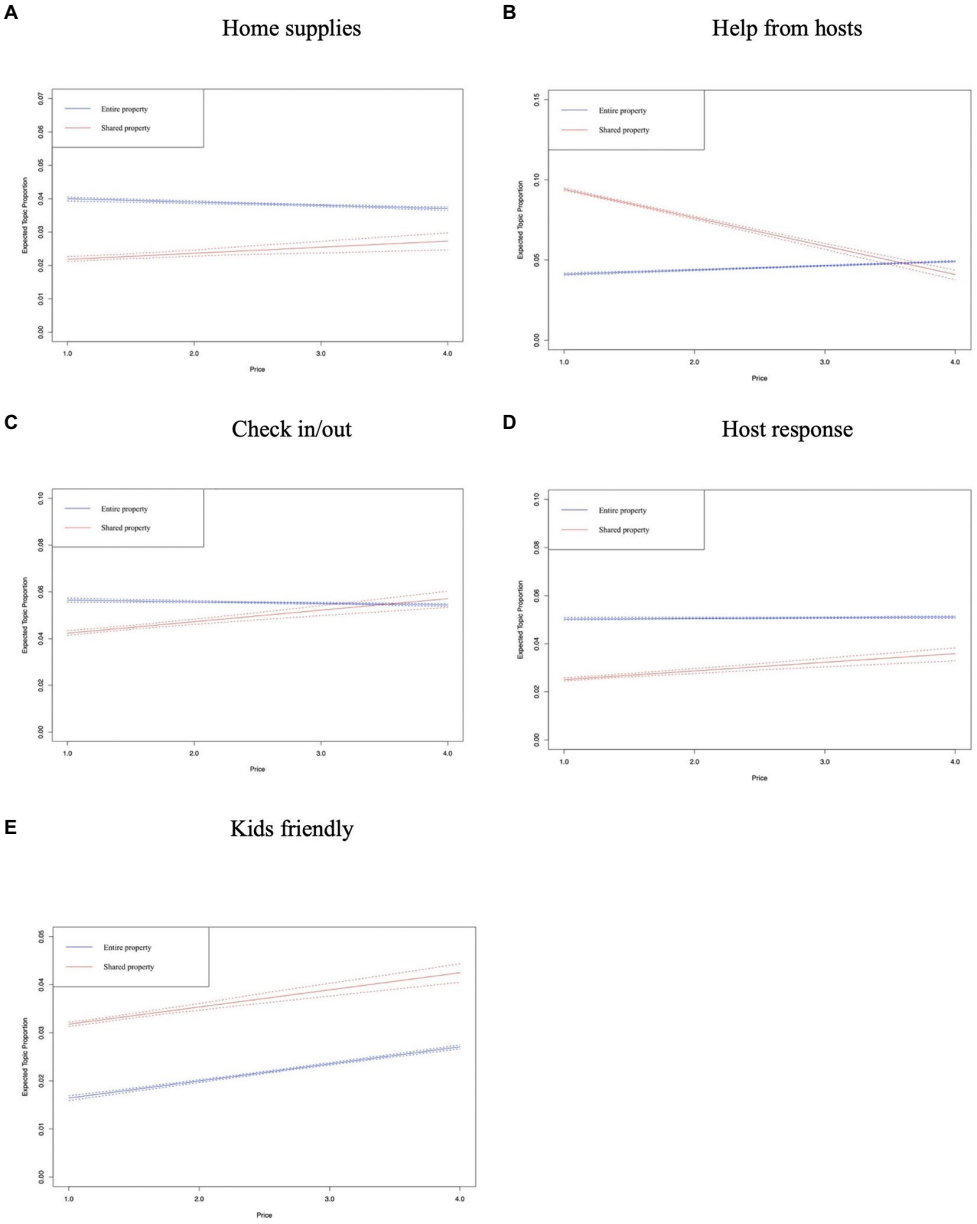
Topics in the dimension of service. **(A)** Home supplies. **(B)** Help from hosts. **(C)** Check in/out. **(D)** Host response. **(E)** Kids friendly.

**Figure 10:**
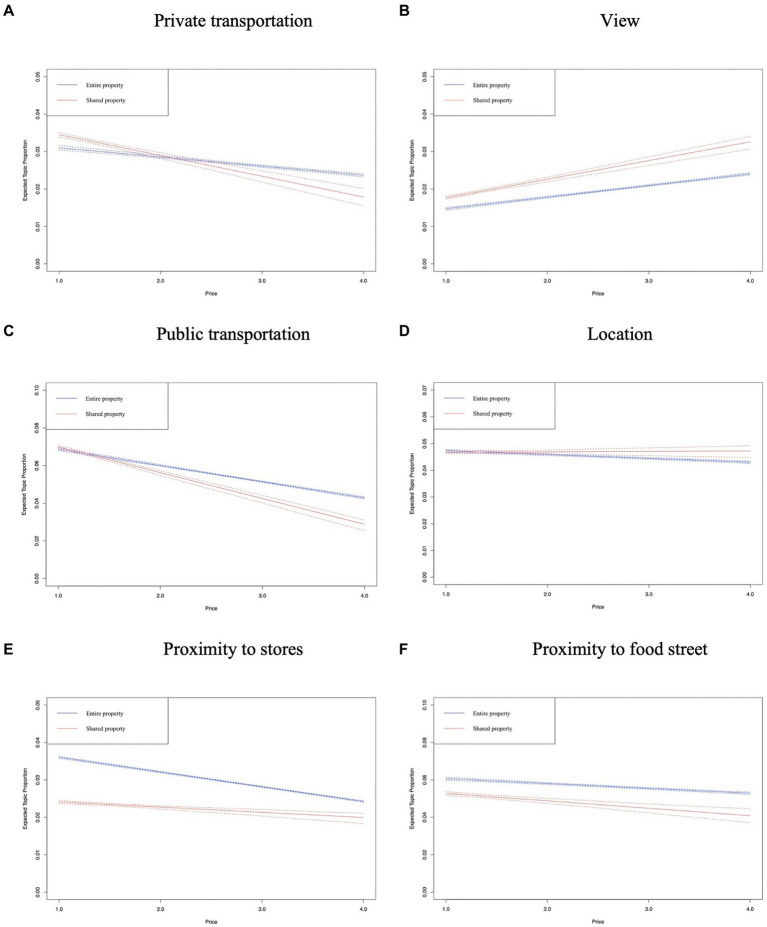
Topics in the dimension of location. **(A)** Private transportation. **(B)** View. **(C)** Public transportation. **(D)** Location. **(E)** Proximity to stores. **(F)** Proximity to food street.

**Figure 11:**
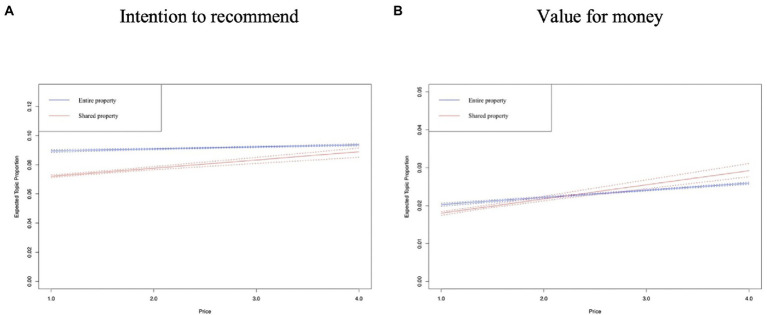
Topics in the dimension of value. **(A)** Intention to recommend. **(B)** Value for money.

**Figure 12:**
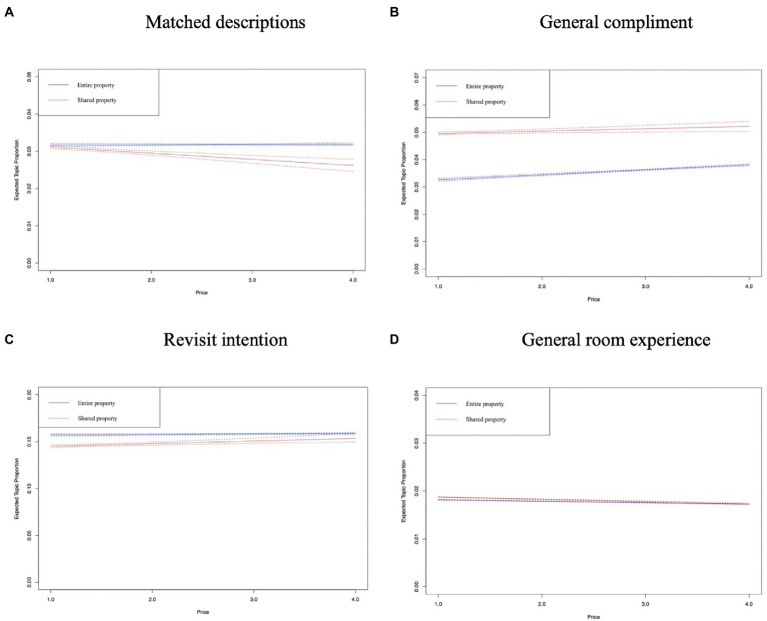
Topics in the dimension of general experience. **(A)** Matched descriptions. **(B)** General compliment. **(C)** Revisit intention. **(D)** General room experience.

In Figures 8–12, the *x*-axis represents the average price of the listing, and the *y*-axis represents the expected topic proportion. The blue and red lines indicate the expected topic proportion for *entire property* and *shared property*.

In the dimension of facility, Figures 8A,C shows that the prevalence of these two topics sees a declining trend in both types of Airbnb accommodation, in particular, the topic of *poor living environment*. The expected proportion of this topic decreases more obviously as the price goes up, which indicates that Airbnb users are more likely to have an unfavorable lodging experience in a relatively inexpensive property. Figure 8B shows that the prevalence of *pool* in *entire property* increases significantly from about 7% (*price* = 1) to 16% (*price* = 4). However, this figure for *shared property* increases slightly with respect to the average daily listing price. Figure 8D shows that the prevalence of *group accommodation* increased moderately with the increase in price in *entire property*, which contrasts with the changing pattern of topic prevalence in *shared property*.

Figures 9A,C,D does not exhibit a very significant change in topic prevalence as the price increased in both types of Airbnb accommodation. Figure 9E shows that the prevalence of *hep from hosts* decreased obviously from around 8% (price = 1) to 4% (price = 4). The potential reason is that Airbnb users who stayed in low-cost *shared property* were more likely to encounter the accommodation problems shown in the representative reviews of this topic, such as non-working air conditioners and clogged drains. Figure 9E shows that, for both types of Airbnb accommodations, the higher the price, the more likely guests are to be concerned about their children’s accommodation experience.

Figures 10A,C–F shows that in both types of Airbnb accommodation, users who stay in relatively inexpensive properties are more concerned about the convenience of exploring the local area, especially regarding the availability of transportation. In Figure 10F, the authors find that the prevalence of *view* rises as the price goes up in both *entire property* and *shared property*, which reveals one of the factors that makes Airbnb users willing to choose a higher-priced property.

According to Figure 11A, the prevalence of *intention to recommend* increases more significantly in *shared property* than in *entire property* as the price increases. Figure 11B shows that more Airbnb users comment on the value for money in higher-priced properties, and the authors found that Airbnb users often compare traditional hotels at the same price to reflect the value for money of the Airbnb accommodation.

Figures 12A–D shows that the proportions of four topics (i.e., *matched descriptions*, *general compliment*, *revisit intention*, and *general room experience*) in both types of accommodations remain evenly distributed.

Based on the above analysis, it can be concluded that the emphasis of Airbnb users on service attributes is different when they stay at *entire property* and *shared property*. Guests staying at *entire property* may have more interaction with the host, as evidenced by the fact that the topic of *help from hosts* appears more often in *entire property*, indicating the important role of hosts in guests’ lodging experiences. Guests staying at *shared property* care more about the convenience of exploring the places of interest, such as stores and food streets. Airbnb users’ evaluation of accommodation services also shows significant differences with the change in listing prices. There are also some similarities between Airbnb users staying at *entire property* and *shared property*. As listing prices increase, Airbnb users are more likely to care about the surroundings (i.e., the view) than the convenience associated with the location. In addition, Airbnb users who stay at both types of accommodations are concerned with the experience of their children.

## 5. Conclusion

### 5.1. Discussion

This study compared the perceptions of Airbnb users staying at Airbnb accommodations with different levels of sharing and price ranges. The results indicate the distinctive preferences of Airbnb users staying at *entire property* and *shared property*. For those staying at *entire property*, they are less money sensitive. In contrast, Airbnb users staying at *shared property* pay more attention to the economic value, which is in line with Xu’s (2020) findings. Another notable finding of the comparative analysis is that Airbnb users who stay at *shared property* and *entire property* have very distinct differences in their interactions with hosts during the accommodation phase. Airbnb users who stayed at *entire property* highlighted the interaction throughout the stay, including assistance with check-in, problem-solving during the stay, and help at check-out. However, Airbnb users who stayed in *shared property* emphasized the interaction during the check-in and check-out phases, and the interaction was mainly related to the efficiency of communication, with less dependence on the host. Despite the fact that Airbnb users who chose different types of Airbnb accommodation may have different expectations of Airbnb hosts, quality communication with the host is valued by all the users. In previous Airbnb studies, the important role of Airbnb hosts’ responsiveness has been widely confirmed (Ju et al., 2019; Zhuo and Wang, 2022). In the present study, through the analysis of example reviews, we found that users mainly evaluate the quality of communication with their hosts in two ways, namely, the efficiency of the response and the usefulness of the response content.

In line with Chiu and Chen (2014) and Rhee and Yang (2015), the findings of this study support that customers’ emphasis on product and service attributes differ in accommodation with different price ranges. In this study, those who stay at relatively low-cost Airbnb accommodations are more concerned about accessibility, such as easy access to public transportation, proximity to stores, and proximity to food courts. However, those staying at relatively higher-priced accommodations are more concerned about enjoyment-related attributes such as pools, children’s play facilities, and views of the surrounding area. In addition, in both types of Airbnb accommodations, Airbnb users are quite concerned about the convenience of exploring the surrounding areas of interest as prices drop, as evidenced by the change in topics related to transportation and location. This finding provides further evidence that Airbnb users tend to look for a more authentic local experience at an affordable price (Priporas et al., 2017). Regarding customers’ perception of value for money, the finding of this study is inconsistent with results derived from traditional hotels. Budget hotel customers were generally found to attach more importance to value for money (Rahimi and Kozak, 2017; Kim et al., 2019). However, we found that Airbnb users staying at relatively higher-priced Airbnb accommodations paid more attention to price value. This difference could be attributed to the nature of the P2P accommodation business, which aims to provide travelers with a value-for-money accommodation solution; hence, Airbnb users who pay higher prices may care more about value for money. Therefore, Airbnb hosts who operate high-priced properties should ensure that the price their customers pay matches the service provided.

The results of topic correlation analysis explicitly revealed relationships between different topics, and most of the connections between these topics are interpretable. It is notable that the topic of *host response* is both connected with *intention of recommend* and *revisit intention*, and *pool* is also connected *with intention of recommend*, which reveals factors that can contribute to Airbnb users’ loyalty behaviors. This finding is consistent with Wang and Jeong’ (2018) finding that Airbnb users’ satisfaction is affected by amenities and the host–guest relationship, which can lead to loyal customers and repeat business.

### 5.2. Theoretical implications

As for theoretical implications, this study extends the body of knowledge on the impact of price on consumers’ perceptions of services by enriching its content in the area of P2P accommodation. Previous studies (Priporas et al., 2017; Ju et al., 2019; Zuo et al., 2022) on Airbnb users’ post-consumer experiences have provided a general understanding of the service and product attributes that Airbnb users care about. This study aimed to extend our understanding of the influence of price on Airbnb users’ evaluations of their lodging experiences using online Airbnb reviews as the data source. Similar to the findings derived from the research on traditional hotels (Knutson et al., 1993; Chiu and Chen, 2014), this study adds more evidence that customers’ perceptions change with the change in accommodation price in the context of P2P accommodation. This study reveals that Airbnb service and product attributes that users care about vary to a greater degree across four different price ranges of Airbnb accommodations. This is the first study to examine the variation in Airbnb users’ perceptions across Airbnb listings in different price ranges, which could provide insight for future research on this aspect of P2P accommodation. This study also extends Xu’s (2020) study on how Airbnb users evaluate service attributes in rooms with different levels of sharing by comparing the prevalence of service attribute-related topics using STM. The same findings in this study as in Xu (2020) include that Airbnb users who stay in *shared property* are more concerned with economic value. In addition, this study also found Airbnb users staying at *entire property* valued communication with hosts more than those staying at *shared property*. Finally, this study examined not only key service and product attribute-related topics but also the interconnection between these extracted topics, which can serve as a useful reference for future studies to investigate Airbnb user behavior. For instance, some topics are related to the loyalty behavior of Airbnb users, i.e., *intention to recommend* and *intention to revisit*, whether this relationship exists can be verified in future studies. From a methodological perspective, this study further demonstrates the suitability of using STMs to process unstructured customer review data, which can provide a more effective and flexible solution to derive insights into the customer experience. Future research could include some other features of Airbnb accommodations in the STM to examine the needs of Airbnb users in different market segments.

### 5.3. Practical implications

In terms of managerial implications, this study provides insights into the improvement of the Airbnb user experience and the development of marketing strategies. There are some tailored suggestions for hosts who operate *entire property* and *shared property*. For hosts who operate *entire property*, attention needs to be paid to interacting with guests throughout their stay, as guests staying at *entire property* are likely to expect more interactions with hosts. For those staying at *shared property* who are able to interact with other guests staying at the same property during their stay, there is less reliance on the host, but the host must maintain timely communication, especially during the check-in process. Through topic correlation analysis, it is found that the topic *host response* is related to both topics *intention to recommend* and *revisit intention*, suggesting that Airbnb hosts’ responses are related to guests’ loyalty behavior. As for marketing insights, in both types of Airbnb accommodations, family guests are concerned about their children’s experiences as the price increases. Therefore, child-friendly features should be highlighted in the listing description for hosts operating higher-priced listings. As for hosts who operate relatively low-priced accommodations, they should focus more on promoting the convenience of exploring the surrounding area, as guests staying at relatively low-priced accommodations are more concerned about the convenience of the location. Considering the diversity of customer needs, we recommend that when analyzing customer needs, Airbnb management should take into account the characteristics of products used by the customers, which will help to accurately grasp the needs of different customers and thus improve their stay experience.

### 5.4. Limitations and future research

This study only considered two product features of Airbnb accommodation, namely, price and accommodation type, which could be insufficient to understand the changes in Airbnb users’ behavior. Future studies are suggested to include more diverse variables to compare how Airbnb users evaluate their lodging experience. Despite the fact that Kuala Lumpur is a tourism hotspot and there is a lack of detailed research on local Airbnb, the analysis of Airbnb data in Kuala Lumpur limits the generality of the results. The authors suggest that future research examining the Airbnb user experience in other cities in developing countries could provide valuable comparative insights into Airbnb user behavior.

## Data availability statement

The data analyzed in this study is subject to the following licenses/restrictions: Restrictions from the third-party data provider (AirDNA). Requests to access these datasets should be directed to dingkai3333@163.com.

## Author contributions

KD: conceptualization, methodology, writing—original draft preparation, software, and visualization. WC: validation and writing—reviewing and editing. KN: software and data curation. QZ: revising and editing. All authors contributed to the article and approved the submitted version.

## Funding

The publication fee is provided by Ningbo University of Finance and Economics.

## Conflict of interest

The authors declare that the research was conducted in the absence of any commercial or financial relationships that could be construed as a potential conflict of interest.

## Publisher’s note

All claims expressed in this article are solely those of the authors and do not necessarily represent those of their affiliated organizations, or those of the publisher, the editors and the reviewers. Any product that may be evaluated in this article, or claim that may be made by its manufacturer, is not guaranteed or endorsed by the publisher.

## Author disclaimer

This research is not affiliated with, sponsored by, or endorsed by Airbnb Inc. The views and opinions expressed in this research are those of the authors and do not necessarily reflect the official policy or position of Airbnb. The authors have conducted this research independently and the content has not been reviewed or approved by Airbnb. Any references to Airbnb are made purely for informational purposes and should not be construed as an endorsement of the company or its services.
